# A predictive assessment of genetic correlations between traits in chickens using markers

**DOI:** 10.1186/s12711-017-0290-9

**Published:** 2017-02-01

**Authors:** Mehdi Momen, Ahmad Ayatollahi Mehrgardi, Ayoub Sheikhy, Ali Esmailizadeh, Masood Asadi Fozi, Andreas Kranis, Bruno D. Valente, Guilherme J. M. Rosa, Daniel Gianola

**Affiliations:** 1 Department of Animal Science, Faculty of Agriculture, Shahid Bahonar University of Kerman (SBUK), Kerman, Iran; 2 Department of Statistical, Faculty of Mathematic and Computer Science, Shahid Bahonar University of Kerman (SBUK), Kerman, Iran; 3 State Key Laboratory of Genetic Resources and Evolution, Yunnan Laboratory of Molecular Biology of Domestic Animals, Kunming Institute of Zoology, Chinese Academy of Sciences, Kunming, 650223 China; 4 Roslin Institute, University of Edinburgh, Midlothian, UK; 5 Department of Animal Sciences, University of Wisconsin, Madison, WI USA; 6 Department of Biostatistics and Medical Informatics, University of Wisconsin, Madison, WI USA; 7 Department of Dairy Science, University of Wisconsin, Madison, WI USA

## Abstract

**Background:**

Genomic selection has been successfully implemented in plant and animal breeding programs to shorten generation intervals and accelerate genetic progress per unit of time. In practice, genomic selection can be used to improve several correlated traits simultaneously via multiple-trait prediction, which exploits correlations between traits. However, few studies have explored multiple-trait genomic selection. Our aim was to infer genetic correlations between three traits measured in broiler chickens by exploring kinship matrices based on a linear combination of measures of pedigree and marker-based relatedness. A predictive assessment was used to gauge genetic correlations.

**Methods:**

A multivariate genomic best linear unbiased prediction model was designed to combine information from pedigree and genome-wide markers in order to assess genetic correlations between three complex traits in chickens, i.e. body weight at 35 days of age (BW), ultrasound area of breast meat (BM) and hen-house egg production (HHP). A dataset with 1351 birds that were genotyped with the 600 K Affymetrix platform was used. A kinship kernel (**K**) was constructed as **K** = *λ* **G** + (1 − *λ*)**A**, where **A** is the numerator relationship matrix, measuring pedigree-based relatedness, and **G** is a genomic relationship matrix. The weight (*λ*) assigned to each source of information varied over the grid *λ* = (0, 0.2, 0.4, 0.6, 0.8, 1). Maximum likelihood estimates of heritability and genetic correlations were obtained at each *λ*, and the “optimum” *λ* was determined using cross-validation.

**Results:**

Estimates of genetic correlations were affected by the weight placed on the source of information used to build **K**. For example, the genetic correlation between BW–HHP and BM–HHP changed markedly when *λ* varied from 0 (only **A** used for measuring relatedness) to 1 (only genomic information used). As *λ* increased, predictive correlations (correlation between observed phenotypes and predicted breeding values) increased and mean-squared predictive error decreased. However, the improvement in predictive ability was not monotonic, with an optimum found at some 0 < *λ* < 1, i.e., when both sources of information were used together.

**Conclusions:**

Our findings indicate that multiple-trait prediction may benefit from combining pedigree and marker information. Also, it appeared that expected correlated responses to selection computed from standard theory may differ from realized responses. The predictive assessment provided a metric for performance evaluation as well as a means for expressing uncertainty of outcomes of multiple-trait selection.

## Background

The increasing availability of genome-wide dense molecular markers [e.g., single nucleotide polymorphisms (SNPs)] has opened new avenues for obtaining additional genetic gain in breeding of elite animals and plants by exploiting “genomic selection” methods. These techniques have become important tools in modern breeding programs [1, 2]. Many statistical methods with parametric or non-parametric formulations have been proposed to predict either genomic estimated breeding values (GEBV) of animals or yet-to-be observed phenotypes [1, 3–6].

Most prediction studies have been based on single-trait (uni-variate) statistical models. However, in practice, animals and plants often must be evaluated for several economically important traits. Multiple-trait model predictions have been typically regarded as better than uni-variate predictions [7]. For example, milk yield and composition in dairy cattle or grain yield and resistance to disease in plants are often analyzed with multiple-trait methods [8, 9]. Multi-trait models based on pedigree information represent the typical modeling strategy used to capitalize on genetic evaluation of several correlated traits before genomic selection methods became popular [10]. A multiple-trait analysis requires knowledge of phenotypic and genetic correlations among characters [7]. These correlations indicate the extent to which measurements on one trait inform about other traits [11], and predictions based on single-trait models do not exploit the extra information provided by other traits.

Multiple-trait genomic selection models (MT-GS) have been explored and tested in research only to a limited extent [12]. A genome-based multiple-trait analysis may also offer insight into mechanisms that create trait associations, such as pleiotropy and linkage disequilibrium (LD) between quantitative trait loci (QTL) and markers [13]. One hypothesis is that correlation parameters that are inferred using whole-genome dense molecular markers may give a novel picture of the genetic correlation between traits [14, 15]. However, the sources of genetic and genomic correlations may be distinct [13, 16]. Genomic correlations depend in part on linkage disequilibrium (LD) relationships between markers and QTL, which are unknown, while genetic correlations are in part a function of LD between QTL. Multivariate genome-based models may produce “missing”: situation in which the genetic correlation is undetected by the markers, “excessive”: LD between markers increase the magnitude of the pleiotropy effects of the QTL, or even “spurious”: there is no pleiotropy but LD between markers and/or pairs of QTL may produce pseudo pleiotropy (abbreviated as MES) genetic correlations and, as a consequence, distort expectations about outcomes of multiple-trait selection.

The objective of this study was to infer genetic and genomic correlations between three traits measured in broilers by exploring linear combinations of pedigree-based (genealogical) and marker-based relationship matrices. As advocated by [17], a predictive approach was used to gauge parameter estimates and to provide an empirical test of the extent of genetic association between traits.

## Methods

### Data

The data consisted of records on 1351 birds from a commercial broiler chicken line that had undergone several generations of selection using the traditional multiple-trait genetic evaluations at the Aviagen Ltd Company (Aviagen Ltd, Newbridge, UK). The traits considered were body weight at 35 days of age (BW), ultrasound area of breast meat (BM), and hen-house production (HHP, total number of eggs laid between weeks 28 and 54). Some features of the dataset and pedigree information are in Table 1. All birds had phenotype records and a known sire and dam, and there were 326 and 274 paternal half-sib and full-sib groups in the sample, respectively. This dataset has also been used in other studies by Abdollahi-Arpanahi et al. [18] and Morota et al. [19].

**Table 1:** Pedigree information and features of the chicken data used

Total birds in the pedigree	1675
Number of sires	326
Number of dams	592
Number of full-sib groups	274
Number of progeny with records and known sire and dam	1351
Number of inbreds (pedigree-based inbreeding >0)	159
Inbreeding coefficient range all birds in the pedigree (%)	0.4 to 10.9

### Phenotype correction

**Fig. 1:**
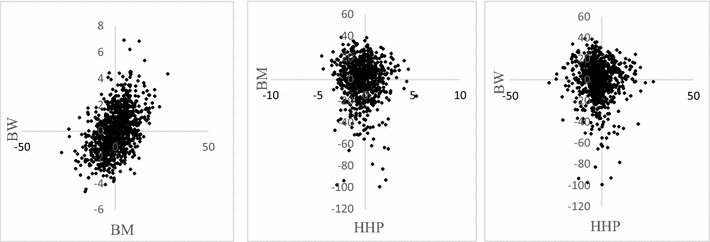
*Scatter plots* of phenotypes pre-corrected for non-genetic sources of variation for body weight (BW), breast muscle area (BM) and hen-house production (HHP)

Prior to implementing the genome-enabled trivariate prediction model, we pre-corrected phenotypes to eliminate all known nuisance non-genetic sources of variation. This correction was based on uni-variate mixed effects models; BW and BM were corrected for a combined effect of sex, hatch week, contemporary group of parents, and pen in the growing farm. HHP was corrected for random hatch effects, with a general mean as the sole fixed effect. Figure 1 shows a scatter plot of pre-corrected phenotypes for these traits. A positive association between BW and BM is suggested, whereas the scatter plots for the pairs BM-HHP and BW-HHP do not indicate concomitant variation.

### Genotyping

The 1351 birds were genotyped using an 600 K Affymetrix SNP chip. SNPs with a minor allelic frequency (MAF) lower than 1% and a call frequency lower than 0.95 were filtered out. Missing genotypes were imputed locus by locus using the Beagle software version 3.3.2 [20]. After quality control, 354,364 SNPs remained for statistical analyses.

### Whole-genome prediction models

Tri-variate linear models were used for estimating (co)variance components and for predicting genomic breeding values. Such models were an extension of a typical single-trait model with random pedigree or genome-based effects, which can be represented as:

1 $${\mathbf{y}}_{t} = {\mathbf{1}}\mu_{t} + {\mathbf{Zg}}_{t} + {\varvec\epsilon }_{t} ;\quad t = 1,2,3,$$

where, **y**_*t*_ is a vector of *m* × 1 pre-corrected phenotypes for trait *t* (*m* = 1351); *μ*_*t*_ is a general constant and 1 is a vector of ones; **Z** is an incidence matrix (an identity matrix in all cases) that allocates records to breeding values; **g**_*t*_ is a vector of additive genetic effects or of direct genomic breeding values, and $\varvec\epsilon_{t}$ is a vector of residuals for trait *t*. It was assumed that ${\mathbf{g}}_{t} \sim\left( {0,{\mathbf{K}}\sigma_{{{\mathbf{g}}_{t} }}^{2} } \right)$ where $\sigma_{{{\mathbf{g}}_{t} }}^{2}$ is the additive genetic or genomic variance of trait *t*, and **K** (*m* × *m*) reflects a covariance structure that results from the combined use of pedigree and marker information, as described later. Random residuals were assumed to follow a normal distribution $\varvec\epsilon_{t} \sim\,N\left( {0,{\mathbf{I}}_{t} \sigma_{{\epsilon_{t} }}^{2} } \right)$, where **I**_*t*_ is an *m* × *m* identity matrix and $\sigma_{{\epsilon_{t} }}^{2}$ is the residual variance for trait *t*; this term represents variation of pre-corrected phenotypes that is not explained by additive genomic effects. The vectors **g**_*t*_ and $\varvec\epsilon_{t}$ were assumed to be independent. The multi-variate model was:

2 $$\left( {\begin{array}{*{20}c} {{\mathbf{y}}_{1} } \\ {{\mathbf{y}}_{2} } \\ {{\mathbf{y}}_{3} } \\ \end{array} } \right) = \left[ {\begin{array}{*{20}c} {{\mathbf{I}}_{1} } & {\mathbf{0}} & {\mathbf{0}} \\ {\mathbf{0}} & {{\mathbf{I}}_{2} } & {\mathbf{0}} \\ {\mathbf{0}} & {\mathbf{0}} & {{\mathbf{I}}_{3} } \\ \end{array} } \right]\left[ {\begin{array}{*{20}c} {\mu_{1} } \\ {\mu_{2} } \\ {\mu_{3} } \\ \end{array} } \right] + \left[ {\begin{array}{*{20}c} {{\mathbf{Z}}_{1} } & {\mathbf{0}} & {\mathbf{0}} \\ {\mathbf{0}} & {{\mathbf{Z}}_{2} } & {\mathbf{0}} \\ {\mathbf{0}} & {\mathbf{0}} & {{\mathbf{Z}}_{3} } \\ \end{array} } \right]\left[ {\begin{array}{*{20}c} {{\mathbf{g}}_{1} } \\ {{\mathbf{g}}_{2} } \\ {{\mathbf{g}}_{3} } \\ \end{array} } \right] + \left[ {\begin{array}{*{20}c} {\varvec\epsilon_{1} } \\ {\varvec\epsilon_{2} } \\ {\varvec\epsilon_{3} } \\ \end{array} } \right],$$

where, **y**_*t*_, *μ*_*t*_, **Z**_*t*_, **g**_*t*_ and $\varvec\epsilon_{t}$ are as before. The vector of multi-trait additive genetic or genomic breeding values was distributed as $$\left[ {\begin{array}{*{20}c} {{\mathbf{g}}_{1} } \\ {{\mathbf{g}}_{2} } \\ {{\mathbf{g}}_{3} } \\ \end{array} } \right]\sim\,N\left( {0,{\mathbf{K}} \otimes {\mathbf{Q}}} \right)$$, where **K** is a kinship or kernel matrix (described later) and **Q** is the (3 × 3) matrix of pedigree- or marker-based covariances among traits. The multivariate residual distribution was assumed to be $$\left[ {\begin{array}{*{20}c} {\varvec\epsilon_{1} } \\ {\varvec\epsilon_{2} } \\ {\varvec\epsilon_{3} } \\ \end{array} } \right]\sim\,N\left( {{\mathbf{0}},{\mathbf{I}} \otimes {\mathbf{R}}} \right)$$, where **R** is the (3 × 3) residual covariance matrix among traits. The Kronecker product (⊗) notation applies to the residual covariance since all traits were measured on all birds.

### Pedigree-based and whole-genome relationship matrices

In a genomic best linear unbiased prediction model (GBLUP), a genomic relationship matrix (**G**) computed from marker data replaces the pedigree-based matrix (**A**) of standard BLUP applications. The genomic relationship matrix intends to measure the realized fraction of alleles shared, rather than the expected fraction, as is the case for **A** [21, 22]. Genomic relationship matrices can be calculated in different ways (e.g., [23]) but here we used two known alternatives, as described next. First, VanRaden [22] proposed the *m* × *m* matrix ${\mathbf{G}}_{\text{V}} = \frac{{{\mathbf{WW}}^{{\prime }} }}{{2\sum {\text{p}}_{\text{i}} {\text{q}}_{\text{i}} }}$, which renders **G** analogous to the numerator relationship matrix **A** due to the denominator, 2Σp_i_q_i_. Here, **W** is a *m* × *p* centered matrix of SNP genotype codes 0, 1 and 2 (*p* = 354,364) and p_i_ is the MAF at locus *i*. Second, Forni et al. [21] suggested a modification of the denominator, ${\mathbf{G}}_{\text{F}} = \frac{{{\mathbf{WW}}^{{\prime }} }}{{\left\{ {{\text{trace}}\left[ {{\mathbf{WW}}^{{\prime }} } \right]} \right\}/{\text{m}}}}$, which attempts to attain compatibility of the genomic relationship matrix with **A** when either the average level inbreeding is low or when the number of generations back to the base population is small.

An alternative to using any given **G** is to combine genomic and pedigree information into a single kinship “kernel” matrix (in the sense of [24]). A “kernel” matrix that exploits genealogy information together with marker-based information could potentially capture parts of the genetic covariance among traits that is not accounted for by either **A** or **G** alone. We followed multiple-kernel ideas [25] and used the kernel **K** = *λ* **G** + (1 − *λ*)**A**, where *λ* is a parameter (weight) bounded between 0 and 1, and **G** = **G**_**V**_ or **G**_**F**_. For example, if *λ* = 0, pedigree information “dominates” in the model, which retrieves a traditional pedigree-based BLUP. Our expectation was that a specific combination of **A** and **G** matrices would provide the “best” estimates of parameters, as gauged by prediction of outcomes, as opposed to using either **A** or **G** alone or both, with (co)variance components estimated in training samples. To assess the best value of *λ*, we applied the grid *λ* = (0, 0.2, 0.4, 0.6, 0.8, 1) and evaluated the ensuing predictive abilities over such a grid.

When using marker- and pedigree-based relationship matrices together, scaling of genomic relationship matrices is needed for interpretation of parameters in the context of theory, e.g., in relation to a base population [26]. Estimates of parameters may be distorted if a genomic relationship matrix is not on the same scale as the pedigree-based relationship matrix. A reasonable rescaling may be achieved by using genomic relationship matrices with elements that range between 0 and 2, which are the minimum and maximum values of **A**, respectively. To render **G** on the same scale as **A**, we used the *map minmax*-*function* that is widely used in machine learning, e.g. [27], as follows:

3 $$Gs_{ij} = \frac{{\left( {Gs_{max} - Gs_{min} } \right) \times \left( {G_{ij} - G_{min} } \right)}}{{G_{max} - G_{min} }} + Gs_{min} .$$

Here, *Gs*_*ij*_ is a scaled element of the **G**_**V**_ or **G**_**F**_ matrix and *G*_*ij*_ is typical element of **G**_**V**_ or **G**_**F**_; *Gs*_*max*_ = 2 and *Gs*_*min*_ = 0 are the minimum and maximum values elements that the scaled matrix is allowed to take, respectively, and *G*_*min*_ and *G*_*max*_ are the maximum and minimum entries of the **G**_**V**_ or **G**_**F**_ matrix, respectively. While **G**_**V**_ and **G**_**F**_ may contain negative off-diagonals, this is not the case for the scaled matrices used here.

### Model fitting and validation

Variance and covariance components were estimated with multiple-trait restricted maximum likelihood (REML) via an average information algorithm (AI) implemented in the WOMBAT program [28]. The software provides point estimates of (co)variance components and their asymptotic standard errors. Matrix **K** = *λ* **G** + (1 − *λ*)**A** was used as kinship matrix, where **G** was either the unscaled or scaled versions **G**_**V**_ or **G**_**F**_.

We used a cross-validation scheme with 20 randomly constructed training and testing sets to assess predictive ability over the grid of *λ* values. We randomly partitioned the whole data into training (60%) and testing (40%) sets in each of the 20 repetitions. After a model was fitted to the training set data, we compared its predictions against realized values in the test set. Predictive ability was measured by mean squared error (MSE) and by the correlation between predicted and observed phenotypes in the testing set.

### Realized versus expected genetic regressions between traits

We also evaluated predictive relationships between pairs of traits, i.e., BW-BM, BW-HHP and BM-HHP, according to the cross-validation scheme described earlier. Over the predefined grid *λ* = (0, 0.2, 0.4, 0.6, 0.8, 1), we computed least-squares estimates of the regression of the phenotype for trait *x* on DGV for trait *y*, and vice versa, for each pair of traits for each of the 20 validation sets. These realized regressions were compared to expected genetic regressions deduced from REML (co)variance component estimates as:

$$b_{{\left( {x,y} \right)}} (\lambda ) = r_{{G\left( {x,y} \right)}} (\lambda ) \times \sqrt {\sigma_{G\left( x \right)}^{2} (\lambda )} /\sqrt {\sigma_{G\left( y \right)}^{2} (\lambda )} ,$$

where, $r_{{G\left( {x,y} \right)}} \left( \lambda \right)$ is the estimated genetic correlation between traits *x* and *y*, and $\sigma_{G\left( x \right)}^{2} \left( \lambda \right)$ and $\sigma_{G\left( y \right)}^{2} \left( \lambda \right)$ are the genetic variances estimated by REML over the predefined grid of *λ*.

## Results

### Heritability

Table 2 shows the heritability estimates obtained for each *λ* value, both for unscaled and scaled genomic relationship matrices. A low to moderate heritability was found for BW, BM and HHP. When using pedigree-based information only, heritability estimates (standard errors in parenthesis) were 0.187 (0.049), 0.244 (0.052) and 0.315 (0.074), respectively (Table 2). These estimated heritabilities changed to 0.165 (0.039), 0.255 (0.042) and 0.196 (0.052) with an unscaled **G**_**F**_, and to 0.156 (0.06), 0.243 (0.04) and 0.174 (0.04) with an unscaled **G**_**V**_. Scaling the genomic relationship matrices increased heritability estimates relative to those obtained from unscaled matrices. Estimated heritabilities in the present study were lower than in [29, 30] using the same population from which our dataset was drawn but with a larger sample size from four generations of three commercial lines, at varying intensities of selection in the Aviagen UK breeding program. For example, in [29, 30] estimates for BW ranged from 0.326 (0.011) to 0.399 (0.015), whereas in our study they ranged from 0.156 (0.06) to 0.187 (0.049). Our result is based on a small subset of birds taken from the overall population; therefore it is expected that estimated heritabilities *h* ^2^ would differ from those obtained using all available data, which would account for past selection.

**Table 2:** Estimates of heritability for body weight (BW), ultrasound area of breast meat (BM) and hen-house egg production (HHP) obtained by placing varying weights (*λ*) on the pedigree-based relationship matrix (**A**) and on Forni’s (**G**_**F**_) or VanRaden’s (**G**_**V**_) relationship matrix

Regularization parameter (*λ*)	**G**_**F**_						**G**_**V**_					
	Unscaled			Scaled			Unscaled			Scaled		
	$h_{BW}^{2}$	$h_{BM}^{2}$	$h_{HHP}^{2}$	$h_{BW}^{2}$	$h_{BM}^{2}$	$h_{HHP}^{2}$	$h_{BW}^{2}$	$h_{BM}^{2}$	$h_{HHP}^{2}$	$h_{BW}^{2}$	$h_{BM}^{2}$	$h_{HHP}^{2}$
**A** (*λ* = 0)	0.187	0.244	*0.315*	0.187	0.244	0.315	0.187	0.244	*0.315*	0.187	0.244	*0.315*
*λ* = 0.20	0.226	0.291	0.309	0.234	0.297	0.340	0.223	0.291	0.299	0.230	0.295	0.311
*λ* = 0.40	*0.232*	*0.303*	0.285	0.278	0.348	0.360	*0.227*	*0.299*	0.270	*0.247*	0.318	0.295
*λ* = 0.60	0.219	0.297	0.258	0.315	0.395	0.374	0.213	0.290	0.239	0.245	*0.323*	0.272
*λ* = 0.80	0.197	0.281	0.230	*0.335*	0.431	*0.377*	0.189	0.272	0.209	0.227	0.316	0.245
**G** (*λ* = 1)	0.165	0.255	0.196	0.313	*0.442*	0.361	0.156	0.243	0.174	0.193	0.293	0.214

The largest estimates are italics

Here, we used scaled kinship matrices to obtain “genetic parameters” which do not necessarily correspond to only those from standard pedigree-based additive genetic relationships or realized genomic pairwise similarities. Following VanRaden [22], if the expectation of **G** is **A**, then; E(**K**) = E(λ**G** + (1 − λ)**A**) = **A**. However, if one uses a scaled **G**_**V**_, it follows from the scaling formula that *E*(*Gs*_*ij*_) = 2*E*(*G*_*ij*_ × *G*_*min*_/(*G*_*max*_ − *G*_*min*_)). The latter expectation cannot be written in a closed form, because this requires knowledge of the distributions of *G*_*min*_ and *G*_*max*_.

Our multiple-trait GBLUP analysis indicated that the highest heritability estimates were not obtained at the extremes (0 or 1) of the *λ* grid. For example, the highest genomic heritability for BW was obtained at *λ* = 0.4(0.8), for unscaled (scaled) **G**_**F**_, and at *λ* = 0.4 for the two versions of **G**_**V**_. Scaling **G**_**F**_ and **G**_**V**_ always increased heritability estimates. For BM, higher heritabilities were obtained when scaling was applied. More specifically, the highest estimates were obtained at *λ* = 1 (**G**_**F**_) and *λ* = 0.6 (**G**_**V**_). With unscaled matrices, the highest heritabilities were obtained at *λ* = 0.4 and *λ* = 0.2. For HHP, the highest heritabilities were found at the extreme values of *λ*: *λ* = 0 for unscaled **G**_**F**_ and **G**_**V**_ (scaled or unscaled) and *λ* = 1 for scaled **G**_**F**_. Adding genomic information had little impact on heritability estimates of HHP, except with scaled **G**_**F**_.

Our findings illustrate a fairly obvious point made by Legarra et al. [26]: genomic heritability and its estimates are not invariant with respect to how **G** is constructed. Hence, inferences and comparisons between results from different studies must be done with care. In short, our results with a multiple-trait model indicated that a pedigree-marker based kernel (**K**) had an impact on heritability estimates and that scaling of the genomic relationship matrix led to higher “heritability” estimates, especially for **G**_**F**_.

### Genetic correlations

**Fig. 2:**
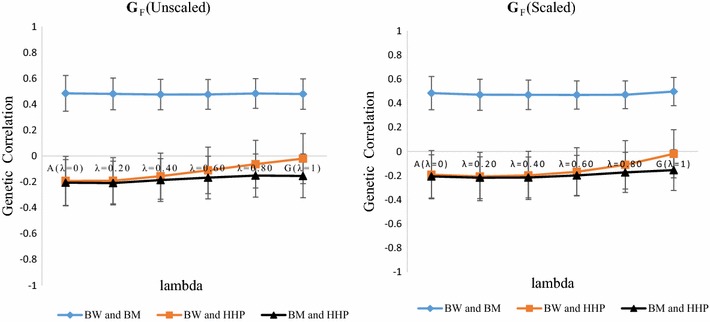
Average and standard errors estimates of genetic and genomic correlations across 20 replicates between body weight (BW), breast meat (BM) and hen-house production (HHP) as a function of the weight placed on Forni’s genomic relationship matrix **G**_**F**_ (*λ*)

**Fig. 3:**
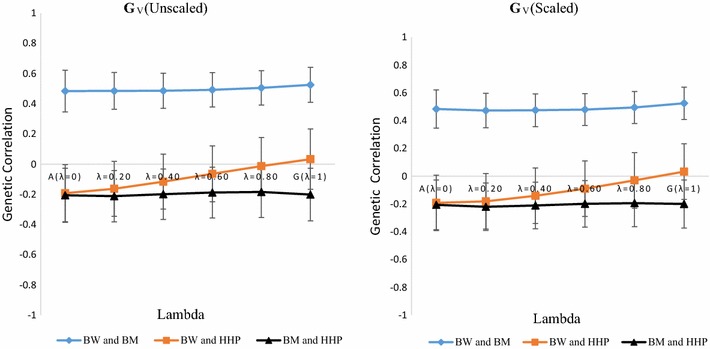
Average and standard errors estimates of genetic and genomic correlations across 20 replicates between body weight (BW), breast meat (BM) and hen-house production (HHP) as a function of the weight placed on VanRaden’s genomic relationship matrix **G**_**V**_ (*λ*)

Estimates of correlations are in Table 3 and Figs. 2 and 3. Estimates of residual and phenotypic correlations were less sensitive to *λ* than genetic correlations, so our discussion concentrates on the latter. All parameters were estimated for each *λ* and for each of the two genomic relationship matrices. When using a pedigree-marker based kinship matrix (**K**), estimates of genetic correlations for BW-HHP and BM-HHP changed gradually when *λ* increased from 0 to 1. Results are shown graphically in Figs. 2 and 3 for **G**_**F**_ and **G**_**V**_, respectively. Changes were more pronounced for the genetic correlation between BW and HHP, which decreased in absolute value from −0.192 (*λ* = 0) to −0.02 (**G**_**F**_, unscaled), −0.019 (**G**_**F**_, scaled), and 0.033 (**G**_**V**_ scaled or unscaled) with *λ* = 1. Estimates of the genetic correlation between BM and HHP were always negative and tended to decrease in absolute value as *λ* increased. They decreased from about −0.206 when only pedigree-based information (*λ* = 0) was used to −0.154 when only genomic information (*λ* = 1) was used to construct **K** from scaled or unscaled versions of **G**_**F**_. BW and BM presented large positive genetic correlation estimates that ranged from 0.484 with the pedigree-based model to 0.497 (0.525) when only **G**_**F**_ (**G**_**V**_) was used. It was insensitive to scaling of the **G** matrix. Standard errors of estimates for BM-BW (results not shown) tended to decrease when *λ* increased. There were no clear tendencies for the standard errors of estimates of the genetic correlation of BW with HHP and BM with HHP. In short, classical genetic correlations (based on **A**) and genomic correlations (based on **G**) were distinct, depending on the pairs of traits considered. However, varying *λ* from 0 to 1 produced very minor changes in estimates of the genetic correlation between BM and HHP, but large changes in estimates of the genetic correlation between BW and HHP. Estimates of the genetic correlations between BW and BM were insensitive to *λ*.

**Table 3:** Phenotypic (*r*_*p*_) and environmental (*r*_*e*_) correlations between body weight (BW), ultrasound area of breast meat (BM) and hen-house egg production (HHP) from a tri-variate analysis with varying weights (*λ*) on the pedigree-based relationship matrix (**A**) and on Forni’s (**G**_**F**_) or VanRaden’s (**G**_**V**_) relationship matrix

Regularization parameter (λ)	$r_{{e \left( {BW,BM} \right)}}$	$r_{{e \left( {BW,HHP} \right)}}$	$r_{{e \left( {BM,HHP} \right)}}$	$r_{{P \left( {BW,BM} \right)}}$	$r_{{P \left( {BW,HHP} \right)}}$	$r_{{P \left( {BM,HHP} \right)}}$
**G**_**F**_						
*Unscaled*						
**A** (*λ* = 0)	0.480 (0.034)	−0.026 (0.058)	−0.010 (0.063)	0.480 (0.023)	−0.066 (0.036)	−0.065 (0.039)
*λ* = 0.20	0.481 (0.036)	−0.023 (0.059)	−0.003 (0.064)	0.479 (0.023)	−0.067 (0.037)	−0.065 (0.039)
*λ* = 0.40	0.482 (0.035)	−0.034 (0.057)	−0.011 (0.061)	0.479 (0.023)	−0.065 (0.037)	−0.063 (0.039)
*λ* = 0.60	0.482 (0.033)	−0.047 (0.053)	−0.020 (0.057)	0.479 (0.023)	−0.062 (0.036)	−0.061 (0.039)
*λ* = 0.80	0.481 (0.031)	−0.058 (0.049)	−0.028 (0.053)	0.479 (0.023)	−0.059 (0.036)	−0.059 (0.038)
**G** (*λ* = 1)	0.479 (0.029)	−0.068 (0.045)	−0.035 (0.049)	0.479 (0.023)	−0.059 (0.036)	−0.062 (0.038)
*Scaled*						
*λ* = 0.20	0.484 (0.035)	−0.022 (0.059)	−0.006 (0.064)	0.479 (0.024)	−0.074 (0.039)	−0.073 (0.041)
*λ* = 0.40	0.484 (0.036)	−0.024 (0.059)	−0.006 (0.063)	0.478 (0.027)	−0.079 (0.042)	−0.080 (0.045)
*λ* = 0.60	0.485 (0.035)	−0.034 (0.057)	−0.012 (0.061)	0.477 (0.031)	−0.080 (0.047)	−0.084 (0.049)
*λ* = 0.80	0.484 (0.033)	−0.050 (0.052)	−0.062 (0.078)	0.477 (0.035)	−0.072 (0.052)	−0.084 (0.055)
**G** (*λ* = 1)	0.479 (0.029)	−0.068 (0.045)	−0.035 (0.049)	0.481 (0.038)	−0.052 (0.056)	−0.082 (0.059)
**G**_**V**_						
*Unscaled*						
*λ* = 0.20	0.480 (0.036)	−0.033 (0.058)	−0.004 (0.063)	0.480 (0.023)	−0.066 (0.037)	−0.066 (0.039)
*λ* = 0.40	0.480 (0.035)	−0.046 (0.055)	−0.011 (0.059)	0.480 (0.023)	−0.064 (0.036)	−0.064 (0.039)
*λ* = 0.60	0.479 (0.033)	−0.060 (0.051)	−0.018 (0.055)	0.480 (0.023)	−0.061 (0.036)	−0.063 (0.039)
*λ* = 0.80	0.476 (0.030)	−0.070 (0.047)	−0.024 (0.051)	0.481 (0.023)	−0.059 (0.036)	−0.062 (0.038)
**G** (*λ* = 1)	0.474 (0.028)	−0.079 (0.044)	−0.030 (0.047)	0.481 (0.023)	−0.060 (0.036)	−0.065 (0.038)
*Scaled*						
*λ* = 0.20	0.484 (0.036)	−0.030 (0.059)	−0.006 (0.063)	0.479 (0.024)	−0.070 (0.037)	−0.071 (0.040)
*λ* = 0.40	0.483 (0.035)	−0.043 (0.056)	−0.010 (0.060)	0.479 (0.024)	−0.069 (0.038)	−0.072 (0.041)
*λ* = 0.60	0.482 (0.033)	−0.057 (0.053)	−0.018 (0.056)	0.479 (0.025)	−0.066 (0.039)	−0.072 (0.041)
*λ* = 0.80	0.478 (0.031)	−0.070 (0.048)	−0.025 (0.051)	0.481 (0.025)	−0.061 (0.039)	−0.072 (0.042)
**G** (*λ* = 1)	0.474 (0.028)	−0.078 (0.044)	−0.030 (0.047)	0.483 (0.025)	−0.056 (0.039)	−0.073 (0.042)

**A**: numerator relationship matix, **G**_**F**_: Forni’s relationship matrix, **G**_**V**_: VanRaden’s relationship matrix

The differences that were observed in estimates of genetic correlations depended on the type of information used. From theory, standard pedigree-based linear models capture expected genetic covariation, whereas marker-based models capture genetic covariation that is marked by SNPs. Our results are important from the perspective of multiple-trait genomic analysis because they indicate that estimates of genetic correlations between some traits may depend on the type of information used. This was clearly the case for the genetic correlation between BW and HHP.

Multiple-trait pedigree or marker-based prediction was designed to exploit genetic correlations between target characters and indicator traits [16], especially when a lowly heritable target trait is genetically correlated with an indicator that has a higher heritability. Our results indicate that estimates of genomic correlation between characters may reaffirm or disagree with expectations that are developed from a pedigree-based analysis. For example, on the one hand, the genetic and genomic correlations between BW and BM were insensitive to *λ* values, i.e., estimates of the genomic correlation and of the genetic correlation derived from the infinitesimal model were the same. On the other hand, when considering BW and HHP, the estimate of the pedigree-based genetic correlation was equal to 0.2, whereas the estimate of the genomic correlation was close to 0. This illustrates a situation where part of the covariance between a pair of traits was not detected by SNPs (“missing correlation”). Sources of genetic correlation may be lost in a multiple-trait marker-based analysis. In the case of BM and HHP, the classical genetic correlation was estimated at −0.20 and the genomic correlation at −0.15. The pedigree-based analysis suggested a stronger genetic correlation.

Care should be exercised when interpreting and using genetic parameters that are assessed via molecular markers, as predictions for complex traits based on pedigree data may differ significantly from those based on SNP data. For this reason, we explored whether the two sources of information could be combined in some “optimal” manner.

### Predictive ability

**Fig. 4:**
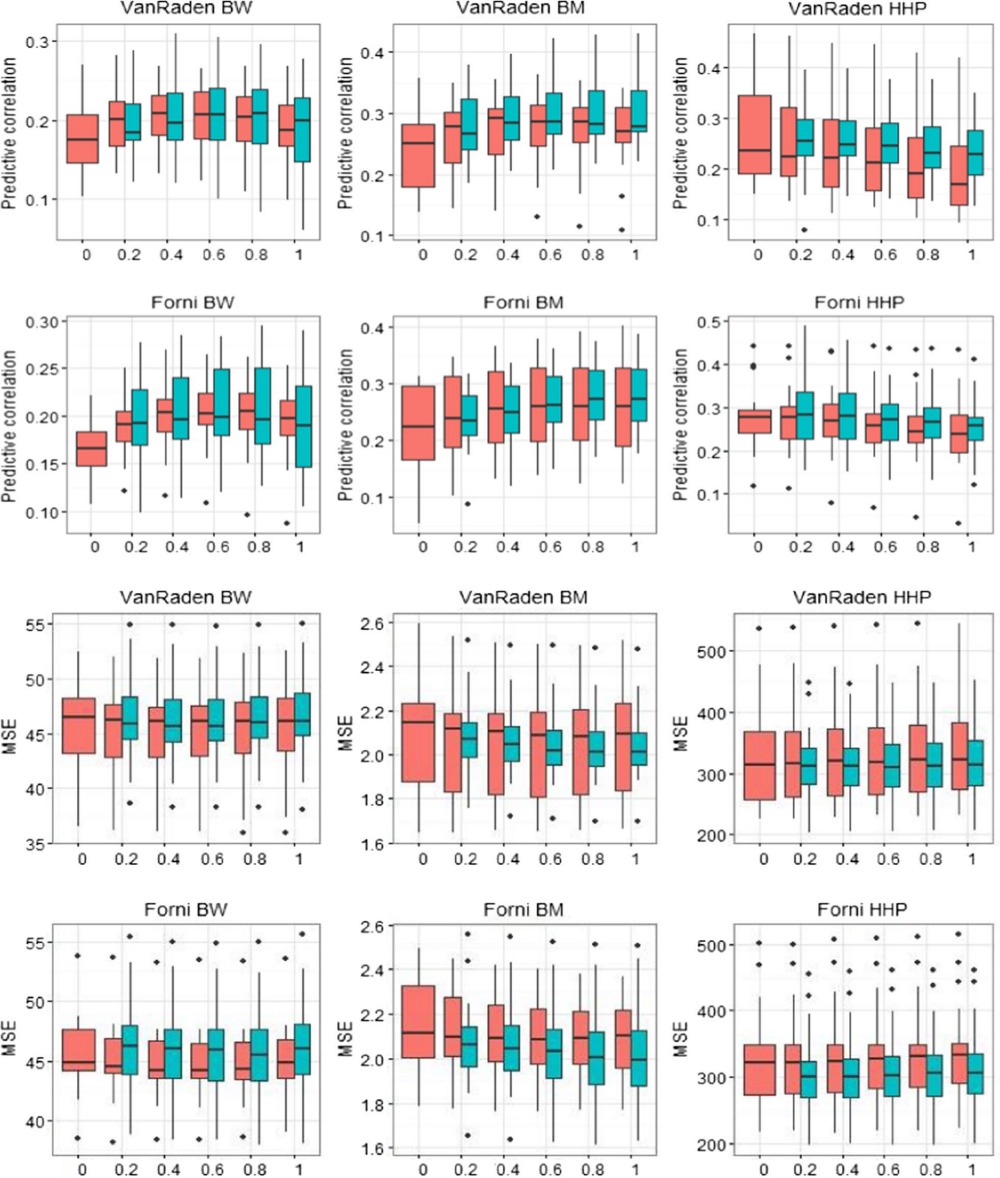
*Boxplot* of predictive correlations across 20 replicates between phenotypes and predicted breeding values (*upper two rows*), and of mean squared errors (MSE) (*bottom two rows*) in testing sets. *Red* and *light blue colors* denote values for unscaled and scaled relationship matrices of Forni or VanRaden, respectively. Outliers are denoted as *black dots*, and the *x*-axis label denotes λ = 0, 0.2, 0.4, 0.6, 0.8, 1

The question of how to arrive at a “best” estimate of a genetic correlation (i.e., for which the greatest advantage of predicting ability is obtained) was examined and, to accomplish this objective, we used the predictive approach advocated by Lo et al. [17]. Figure 4 shows boxplots with the distributions of predictive correlations and mean squared errors for the cross-validation with 20 random repetitions. Some of the plots (e.g., BW) show a mild advantage of using a linear combination of **G** and **A** as kinship kernel. For BW, the largest correlation and lowest MSE were obtained with unscaled **G**_**F**_ and **G**_**V**_. In terms of the predictive correlation for BW, the largest values were obtained with scaled **G**_**V**_ and unscaled **G**_**F**_, both at *λ* = 0.8. For BM, the largest predictive correlation was achieved with unscaled **G**_**V**_ and scaled **G**_**F**_, at *λ* = 0.4 and 0.8, respectively. For HHP, both scaled **G**_**V**_ and **G**_**F**_ resulted in better performance, and the largest predictive correlations were obtained at *λ* = 0.2.

The lowest MSE for BW was achieved for unscaled **G**_**F**_ and scaled **G**_**V**_ at *λ* = 0.6. For BM, the lowest MSE was obtained with *λ* close to 1 using scaled **G**_**V**_ and **G**_**F**_. In addition, the scaled **G**_**V**_ and **G**_**F**_ produced the lowest MSE for HHP, with a slight superiority for values of *λ* close to 1. In terms of MSE, except for BW with **G**_**F**_, scaling of genomic relationship matrices yielded better results. Our findings are in agreement with Rodríguez-Ramilo et al. [31], who reported that when a larger weight was assigned to the numerator relationship matrix (**A**), the predictive correlation was lower than when assigning more weight to the genomic relationship matrix (**G**); a similar behavior was found for MSE. Rodríguez-Ramilo et al. [31] estimated *λ* by using Bayesian methods and reported that the posterior mean of *λ* depended on training sample size and the trait.

Our results indicate that multiple-trait genome-enabled predictions may be improved in some cases by combining **A** and **G** to quantify kinship. This result may also hold when prediction involves multiple selection lines or crossbred animals. Combining kernels can be viewed as a form of model averaging [25], with markers and pedigree playing complementary roles in prediction, e.g., markers may exploit similarity in state and LD, with **A** informing about similarity by descent.

Our results using dense SNPs (600 K Affymetrix platform) indicate that GBLUP with scaled or unscaled relationship matrices typically performed better than pedigree-based BLUP. However, in most cases, the largest correlation and lowest MSE were achieved using a linear combination of **A** and **G**.

### Regression coefficients

**Fig. 5:**
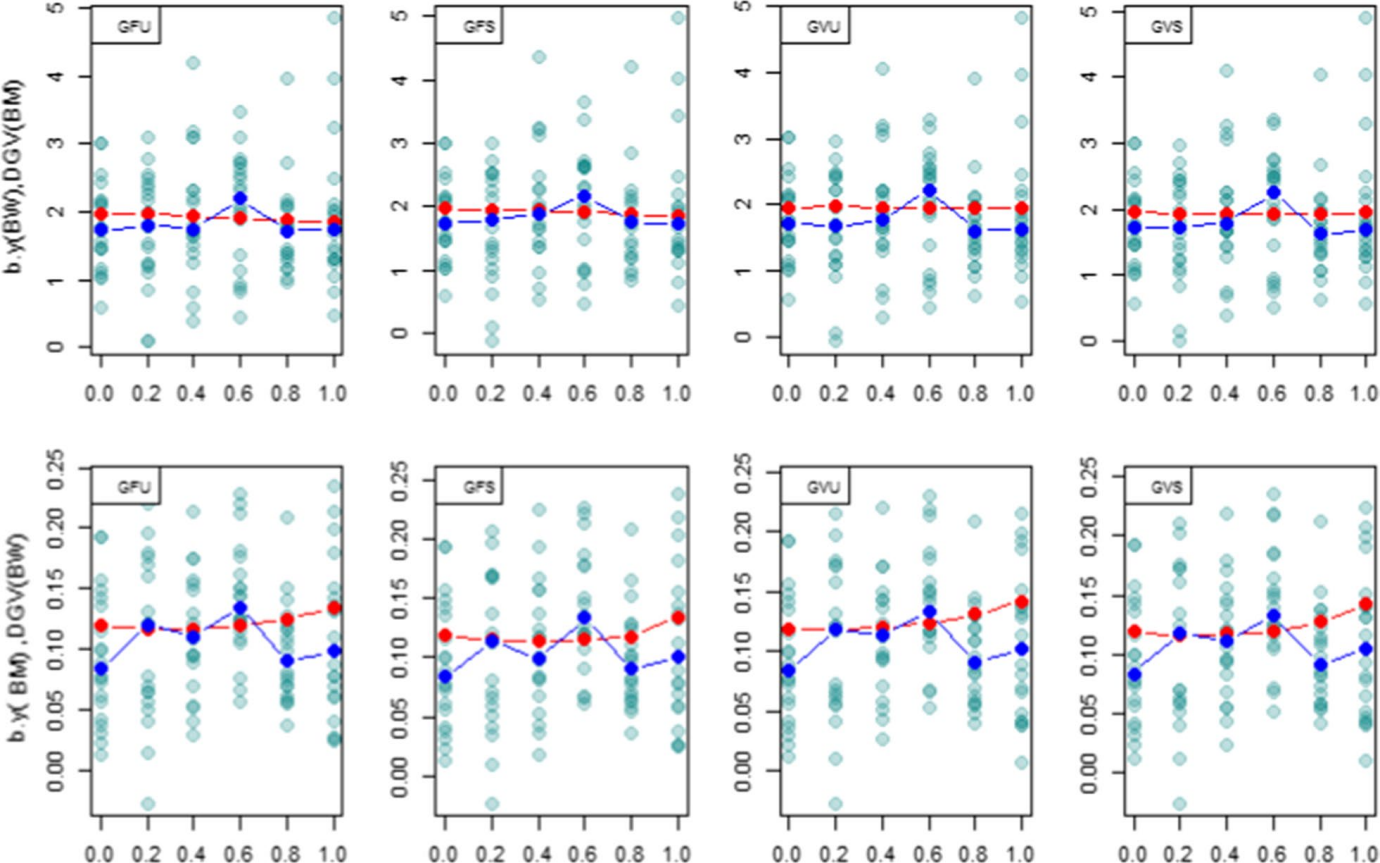
*Scatter plots* of the regression coefficient of observed phenotype for BW on DGV of BM; $b_{{y\left( {BW} \right),DGV\left( {BM} \right)}}$ (*first row*), and the regression of observed phenotype for BM on DGV of BW; $b_{{y\left( {BM} \right),DGV\left( {BW} \right)}}$ (*second row*) in the testing set for 20 cross-validated (CV) regression coefficients. The *red dots* are expected genetic regressions from REML analyses conducted at each *λ*. The *x*-axis label denotes *λ* = 0, 0.2, 0.4, 0.6, 0.8, 1. DGV: direct genomic values; GFU: unscaled Forni’s **G**; GFS: scaled Forni’s **G**; GVU: unscaled VanRaden’s **G**; GVS: scaled VanRaden’s **G**. *Dark blue* points show the median of regressions for 20 random samples

**Fig. 6:**
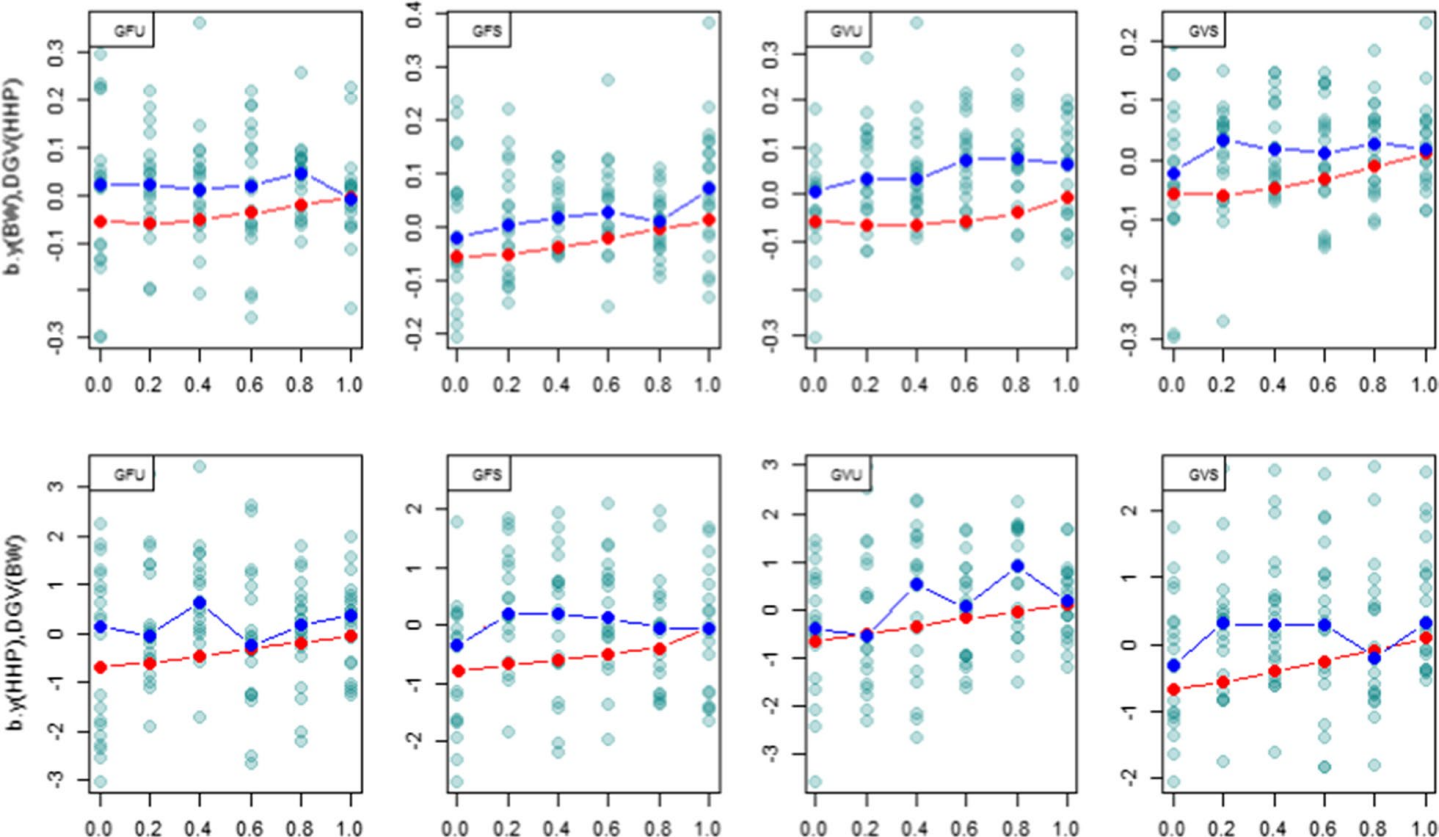
*Scatter plot* of the regression coefficient of observed phenotype for BW on DGV of HHP; $b_{{y\left( {BW} \right),DGV\left( {HHP} \right)}}$ (*first row*) and the regression of observed phenotype for HHP on DGV of BW; $b_{{y\left( {HHP} \right),DGV\left( {BW} \right)}}$ (*second row*) in testing set for 20 cross-validated (CV) regression coefficients. The *red dots* are expected genetic regressions from REML analyses conducted at each *λ* = (0, 0.2, 0.4, 0.6, 0.8, 1). The *x*-axis label denotes *λ* = 0, 0.2, 0.4, 0.6, 0.8, 1. GFU: unscaled Forni’s **G**; GFS: scaled Forni’s **G**; GVU: unscaled VanRaden’s **G**; GVS: scaled VanRaden’s **G**. *Dark blue* points show the median of regression coefficients for 20 random samples

**Fig. 7:**
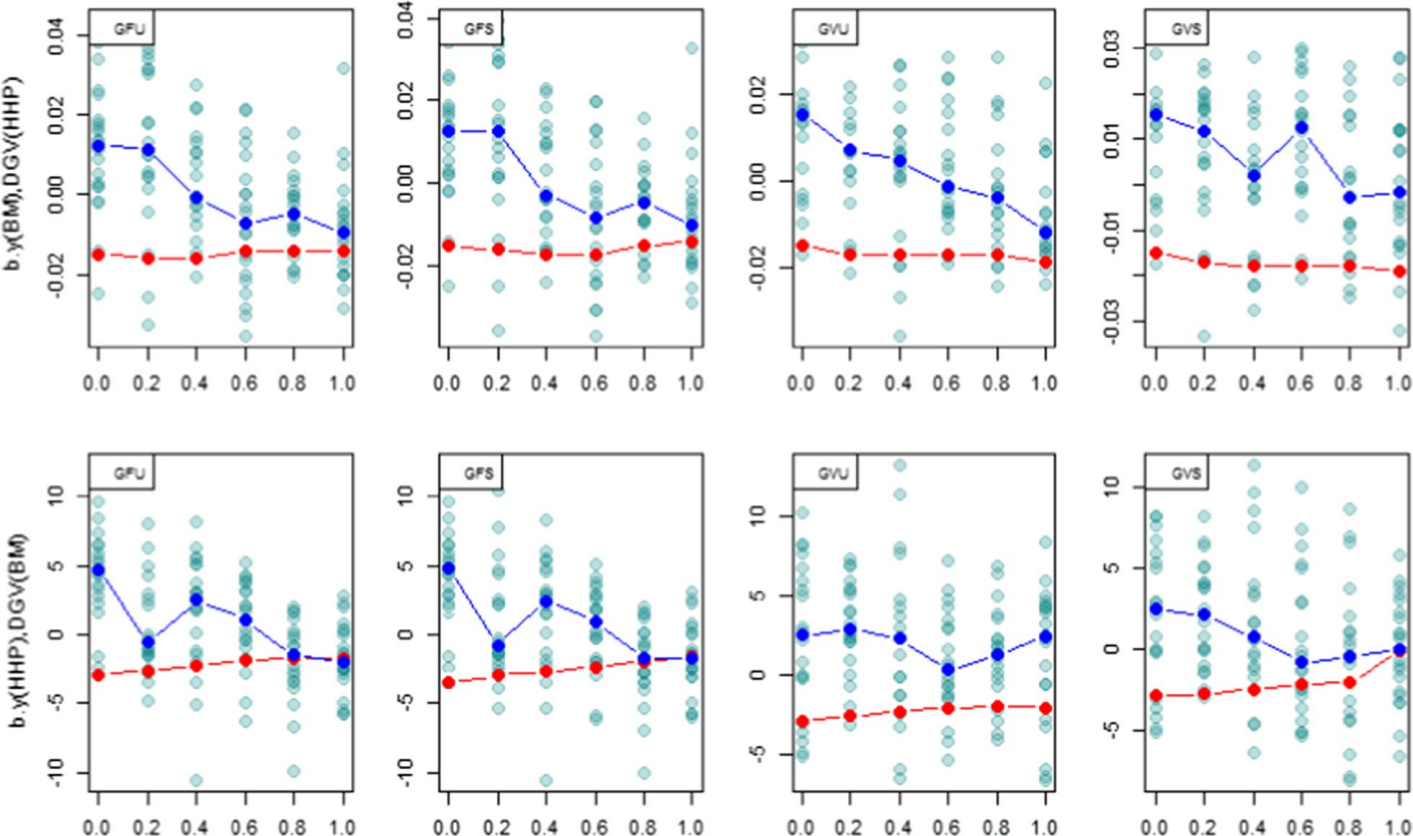
*Scatter plots* of the regression of observed phenotype for BM on DGV of HHP; $b_{{y\left( {BM} \right),DGV\left( {HHP} \right)}}$, (*first row*) and the regression of observed phenotype for HHP on DGV of BW $b_{{y\left( {HHP} \right),DGV\left( {BM} \right)}}$, (*second row*) in testing set for 20 cross-validated (CV) regression coefficients. *Red dots* are expected genetic regressions from REML analyses conducted at each *λ*. The *x*-axis label denotes *λ* = 0, 0.2, 0.4, 0.6, 0.8, 1. GFU: unscaled Forni’s **G**; GFS: scaled Forni’s **G**; GVU: unscaled VanRaden’s **G**; GVS: scaled VanRaden’s **G**. *Dark blue* points show the median of regressions for 20 random samples

Figures 5, 6 and 7 show scatter plots and average (red dotted line) genetic regression coefficients of the three traits on the estimated direct genomic values (DGV) of other traits calculated from REML estimates of (co)variance components. The realized regression coefficients were computed at each *λ* value for the 20 cross-validation random samples and their medians are depicted as dark blue dotted lines on each plot. The REML regressions express the expected change in genetic value of trait *i* if the direct genomic value for trait *j* changes by one unit.

For BW and BM (Fig. 5), the expected and realized regressions were larger than 0 for all values of *λ*. In general, there was reasonable agreement between expected and realized regressions. However, for BW and HHP (Fig. 6), the expected genetic regressions were negative and moved toward 0 as *λ* increased, but the realized regressions (blue dotted lines) varied around 0 for all *λ* values. There was some apparent inconsistency between the expectations based on REML estimates and the cross-validation regression.

Figure 7 indicated a disagreement between expected genetic regressions and cross-validation regressions of BM on HHP when *λ* was close to 0. The expected regressions based on pedigree information were negative, while the cross-validation regressions were positive. The expected regressions of HHP phenotypes on DGV of BM and variances tended towards 0 as *λ* tended to 1, i.e., when more weight was placed on SNPs. The cross-validation regressions were much more affected by the value of *λ* than the expected regressions based on REML estimates.

## Discussion

In genome-enabled prediction, there are different ways of incorporating molecular marker information into parametric and non-parametric models [24, 32]. Research with simulated and real data has consistently shown that single-trait GBLUP displays slightly better prediction accuracy when a trait is affected by a large number of QTL with small effects and as well as other genomic prediction methods for most traits [33, 34]. However, few studies on multiple-trait genomic prediction have been carried out with GBLUP, or have assessed estimates of genetic correlations when genomic or pedigree data were used. Similar to traditional pedigree-based genetic evaluations, the use of multiple-trait GBLUP is expected to increase the accuracy of predictions via “borrowing” of information from genetically correlated traits [35].

In order to explore a multiple-trait GBLUP model that also makes use of pedigree information, we constructed a pedigree-marker based kinship matrix (**K**) as a linear combination of pedigree and marker-based relationships between animals, defined as **K** = *λ* **G** + (1 − *λ*)**A**. Predictive ability of the model and parameter estimates were obtained over a grid of values of *λ* that varied between 0 and 1, e.g., *λ* = 0 implied that all weight was assigned to pedigree, and none to SNPs.

One important factor to take into account when combining marker- and pedigree-based relationship matrices is that such matrices are on the same scale. The elements of the additive relationship matrix are the numerators of Wright’s correlation coefficients that represent the relative genetic variances and covariances among individuals. Consequently, the diagonals of **A** can be as large as 2, and relationships between two individuals can be greater than 1.

Traditionally, to quantify coefficients of relationship with respect to a base (reference) population, as discussed in [36, 37], the probability that alleles are identical by descent (IBD) was derived from pedigree information and from a base population consisting of founders. However, for relationships estimated from genetic markers there is no obvious base population, and they estimate the proportion of the genome that is identical by state (IBS). In our data, genomic relationships measured by unscaled **G**_**V**_ and **G**_**F**_ can take negative values, whereas pedigree relationships are non-negative. In our data, no negative values were observed for full-sib genomic relationships but negative genomic additive relationships with small values near 0 were observed for unrelated individuals based on the pedigree (i.e., pedigree based relationship = 0). It remains to be seen whether genomic relationship measures can detect true ‘negative genomic correlations’ (if such correlations exist), which may be detectable using deep pedigree information and a definition of a base population. The genomic relationship matrices in our analyses were based on (IBS information and on frequencies of alleles to build the GRM.

Our results suggest that multiple-trait genetic predictions depended on the weight assigned to genomic data. Better predictions were often obtained when pedigree and SNP information were used simultaneously. Earlier studies using simulated or real data have explored the potential superiority of multiple-trait over single-trait genomic prediction with a focus on the relationship between traits in terms of differences in heritability, genetic correlations and number of indicator traits (e.g., [35, 38, 39]). De Los Campos et al. [40] indicated that potential problems may emerge when trying to infer genetic parameters using molecular markers that are imperfectly associated with genotypes at causal loci. Gianola et al. [13] showed that correlation parameters that are inferred from markers (i.e., genomic correlations) can give a distorted picture of the genetic correlation between traits. The sources of genetic correlation are pleiotropy (i.e., the same QTL affects more than one trait) and LD between QTL. When markers are used, marker-QTL LD and LD relationships among markers intervene in the genomic correlation.

Here, we examined the impact of combining **A** and **G** on estimates of the genomic correlation between three chicken traits and evaluated outcomes using a predictive framework. Some studies [12, 41] have shown superiority of multiple-trait prediction over single-trait prediction, and combining pedigree with marker information was found to be better than when using either **A** and **G** alone [32].

Our estimates of genetic correlations depended on the choice of *λ*. For example, on the one hand for BW and HHP, when using pedigree as the only measure of similarity (*λ* = 0), the genetic correlation was −0.20, but it shifted to near 0 or was even positive (**G**_**V**_) when only marker information was used. On the other hand, the estimate of the genetic correlation between BW and BM was stable with respect to *λ*, while the estimate of the genetic correlation between BM and HHP was only slightly affected, i.e. changing from −0.21 to −0.15 for **G**_**F**_, and from −0.22 to −0.20 for **G**_**V**_ for *λ* = 0 and *λ* = 1, respectively. Clearly, genomic data provide a distinct measure of similarity between individuals, and this translates into differential capturing of genetic signals. For instance, most off-diagonal entries of **A** were zero but all entries of **G** were non-null.

In order to increase the accuracy of predictions by using pedigree and genomic information jointly, Legarra et al. [42] proposed a single-step procedure that enhances relationship information for non-genotyped animals, without requiring major changes in the implementation of a standard BLUP model. In the study of Aguilar et al. [43], a three-fold increase in accuracy of GEBV was found for traits related to conception rate in Holstein dairy cows, with low heritability, when using a genomic-based relationship combined with a pedigree-based relationship in a multiple trait model [43]. Using 18 quantitative traits in Holstein dairy cattle, Tsuruta et al. [44] reported that prediction accuracies increased when a multiple-trait genomic prediction model was used compared to a single-trait model, but the increase depended on the trait being predicted. However, Bao et al. [45] did not observe clear benefits when four traits were included in a multiple-trait genomic prediction model for soybeans compared to a single-trait model. However, these studies did not examine the impact of using combined genetic and genomic relationships. This shows that the effect of genetic correlations on multi-trait genomic prediction depends on the information type being used to construct **K**, with an impact on accuracy of prediction.

In our study, predictive ability was measured using the correlation between predicted genetic values and observed phenotypic values, and mean squared error of these predictions. As shown in Fig. 4, the optimum weight placed on genomic relationships was trait-dependent. We took the view point that a “best” estimate of the genetic correlation would correspond to the linear combination of **A** and **G** (with a specific weight on each one) that delivered the best predictive ability, which was found by searching the weight (*λ*) placed on genomic versus pedigree relationships. This type of predictive approach has been advocated in the statistical literature [17, 46, 47].

In general, combinations of **A** and **G** kernels yielded better predictions than when only **G** was used. In a GBLUP model, the entries of **G** reflect the actual extent of IBS relationships between individuals, but without making a clear reference to a base population [48]. This implies that genomic (co)variance parameters do not necessarily have the same meaning as standard classical multiple-trait models genetic parameters, such as the infinitesimal genetic correlation. According to [49], pedigree information, co-segregation and population-wide LD are three sources of genetic information that contribute to the predictive ability of genomic selection models. Co-segregation information can be captured by IBD or IBS relationships and, when a pedigree is not deep enough, relatedness among individuals that is inferred from markers may improve prediction. However, how does one decide if an estimate of genetic correlation derived from genomic data is better than a pedigree-based estimate?

In classical quantitative genetics, a genetic correlation between traits arises due to either genes that have an effect on both traits (pleiotropy), or due to LD between genes that affect different traits [50]. When investigating the basis of a genetic correlation, an important question is to determine the extent to which these two forces act on the genetic parameters [13, 51]. Multiple-trait QTL mapping methods may help distinguishing pleiotropy from linkage [52], but any such dissection in the absence of knowledge on QTL would be speculative.

Estimates of genetic correlations obtained from pedigree or from markers may differ either due to chance or other reasons, such as extent of LD between markers and the unknown QTL. One possible way of testing if such differences are systematic, is to examine pairs of estimates of pedigree- and marker-based correlations in re-samples from the dataset and constructing a paired comparison, by using either a parametric or a non-parametric approach. For example, the estimates of correlations could be z-transformed and a paired t test conducted.

In summary, combining pedigree- and marker-based information had an impact on predictive performance of multiple-trait models. Discerning the optimum weight placed on genomic and genealogical information is an important issue, and a grid-search scheme was used for that purpose. We found that estimates of genetic correlation obtained with **A** and **G** matrices were different, but depended on the trait. This indicates that multiple-trait marker-based prediction may be enhanced by the combined use of genealogy and marker information in the models.

## Conclusions

To our knowledge, this is the first study with animal breeding data that explores how the weight placed on pedigree and marker information affects multiple-trait predictions. We designed a tri-variate genomic prediction model that exploited pedigree and marker information simultaneously. Use of a kinship matrix that is formed as a linear combination of pedigree- and marker-based relationships may enhance genome-enabled prediction, but the optimal weight placed on the two sources of information will differ between traits. Genetic correlation estimates from pedigree-based models may differ from those obtained from marker-based models, at least in some cases. Cross-validation was useful for gauging the genetic correlation in multiple-trait models.
